# Health outcomes after stopping long-term mepolizumab in severe eosinophilic asthma: COMET

**DOI:** 10.1183/23120541.00419-2021

**Published:** 2022-01-10

**Authors:** Mark C. Liu, Elisabeth H. Bel, Oliver Kornmann, Wendy C. Moore, Norihiro Kaneko, Steven G. Smith, Neil Martin, Robert G. Price, Steven W. Yancey, Marc Humbert

**Affiliations:** 1 Divisions of Allergy and Clinical Immunology, Pulmonary and Critical Care Medicine, Johns Hopkins Asthma and Allergy Center, Baltimore, MD, USA; 2 Dept of Respiratory Medicine, Amsterdam UMC, University of Amsterdam, Amsterdam, the Netherlands; 3 IKF Pneumologie Frankfurt, Clinical Research Centre Respiratory Diseases, Frankfurt, Germany; 4 Dept of Medicine, Wake Forest School of Medicine, Medical Center Boulevard, Winston-Salem, NC, USA; 5 Dept of Pulmonary Medicine, Kameda Medical Center, Kamogawa, Japan; 6 Respiratory Therapeutic Area, GlaxoSmithKline, Research Triangle Park, NC, USA; 7 Global Medical Affairs, GSK, Brentford, UK; 8 Biostatistics, GSK, Stevenage, UK; 9 Respiratory Therapeutic Area, GSK, Research Triangle Park, NC, USA; 10 Assistance Publique-Hôpitaux de Paris, Service de Pneumologie et Soins Intensifs Respiratoires, Hôpital Bicêtre, Le Kremlin-Bicêtre, Paris, France; 11 Université Paris-Saclay, Paris, France; 12 INSERM U999, Le Kremlin-Bicêtre, Paris, France

## Abstract

Asthma worsening and symptom control are clinically important health outcomes in patients with severe eosinophilic asthma. This analysis of COMET evaluated whether stopping *versus* continuing long-term mepolizumab therapy impacted these outcomes.

Patients with severe eosinophilic asthma with ≥3 years continuous mepolizumab treatment (*via* COLUMBA (NCT01691859) or COSMEX (NCT02135692) open-label studies) were eligible to enter COMET (NCT02555371), a randomised, double-blind, placebo-controlled study. Patients were randomised 1:1 to continue mepolizumab 100 mg subcutaneous every 4 weeks or to stop mepolizumab, plus standard of care asthma treatment. Patients could switch to open-label mepolizumab following an exacerbation. Health outcome endpoints included time to first asthma worsening (composite endpoint: rescue use, symptoms, awakening at night and morning peak expiratory flow (PEF)), patient and clinician assessed global rating of asthma severity and overall perception of response to therapy, and unscheduled healthcare resource utilisation.

Patients who stopped mepolizumab showed increased risk of and shorter time to first asthma worsening compared with those who continued mepolizumab (hazard ratio (HR) 1.71; 95% CI 1.17–2.52; p=0.006), including reduced asthma control (increased risk of first worsening in rescue use (HR 1.36; 95% CI 1.00–1.84; p=0.047) and morning PEF (HR 1.77; 95% CI 1.21–2.59; p=0.003). There was a higher probability of any unscheduled healthcare resource use (HR 1.81; 95% CI 1.31–2.49; p<0.001), and patients and clinicians reported greater asthma severity and less favourable perceived response to therapy for patients who stopped *versus* continued mepolizumab.

These data suggest that patients with severe eosinophilic asthma continuing long-term mepolizumab treatment sustain clinically important improvements in health outcomes.

## Abstract 2

**The COMET study investigated whether stopping long-term mepolizumab had an impact on health outcomes in patients with severe eosinophilic asthma; data suggest those who continue long-term mepolizumab treatment sustain clinically important improvements** https://bit.ly/3A0bvwu

## Introduction

Severe asthma is believed to affect 5–10% of all patients with asthma and encompasses heterogeneous subtypes which either require high-dose controller therapy or are refractory to current therapy [1–5]. Severe eosinophilic asthma is one phenotype, characterised by persistent eosinophilic inflammation and recurrent exacerbations that are not alleviated despite current standard of care [1, 6]. While exacerbations are the primary outcome of many Phase III trials for biologics [7–10] and important in terms of patient welfare and direct costs, further outcomes are also clinically important. Good asthma control is a key treatment goal in asthma management [11], and it is therefore imperative that patients are regularly assessed for symptom control and worsening (indicated by increased rescue medication use, increased symptoms or decreased lung function) [12, 13].

Mepolizumab, a humanised monoclonal antibody that binds to and neutralises interleukin-5, reducing eosinophil proliferation, activation and survival [14, 15], is approved for the treatment of severe eosinophilic asthma and eosinophilic granulomatosis with polyangiitis in multiple regions worldwide and for hypereosinophilic syndrome and chronic rhinosinusitis with nasal polyposis in the USA [14, 16, 17]. Several studies have demonstrated the long-term benefit and tolerability of mepolizumab compared with placebo in patients with severe eosinophilic asthma, showing efficacy in reducing exacerbations, improving lung function, reducing daily oral corticosteroid (OCS) dose and improving health-related quality of life (HRQoL) [18–24].

Further long-term benefit was demonstrated in the randomised, placebo-controlled COMET trial (NCT02555371), which showed that patients with severe eosinophilic asthma who stopped long-term (≥3 years) mepolizumab treatment had a 61% increased risk of experiencing their first clinically significant exacerbation and an approximate six-fold increase in blood eosinophil counts compared with those who continued mepolizumab [10].

In addition to frequent exacerbations, patients with severe eosinophilic asthma typically suffer from ongoing asthma symptoms, which impact day-to-day HRQoL and are associated with high indirect costs [13, 25]. While symptom control, asthma worsening, HRQoL and the patient's perception of disease severity and response to therapy were not primary outcomes in COMET, they are of paramount importance to patients in the clinic [21]. During COMET, the impact of stopping long-term mepolizumab therapy on asthma worsening, patient and clinician perception of disease severity and response to therapy, as well as healthcare resource utilisation (HCRU) were analysed, and the results of the analysis of these clinically important health outcomes are presented here.

## Methods

### Study design

COMET (GSK ID 201810; NCT02555371) has been described previously in detail [10]. Briefly, COMET was a randomised, double-blind, placebo-controlled study that compared stopping *versus* continuing long-term mepolizumab treatment in patients with severe eosinophilic asthma, comprised of four parts (supplementary figure 1). Upon entering the double-blind treatment period (Part C), patients were randomised 1:1 to receive either mepolizumab 100 mg subcutaneous (SC) or placebo SC once every 4 weeks, in addition to standard of care asthma treatment. Patients could switch to open-label mepolizumab (Part D) following an exacerbation. Patients remained on a stable standard of care asthma therapy during Part C to the end of the study.

**FIGURE 1:**
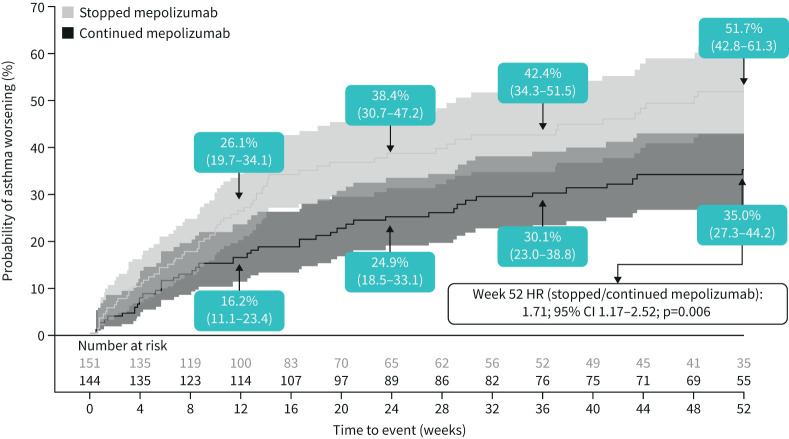
Cumulative incidence of asthma worsening (composite endpoint). Asthma worsening defined as patients meeting at least two of the possible four eDiary criteria for at least two consecutive days; values in boxes indicate the Kaplan–Meier estimates of the probability of asthma worsening with the 95% confidence intervals (also reflected by shaded intervals). Hazard ratio estimated using a Cox proportional hazards model with terms for treatment group and adjustment for baseline covariates. HR: hazard ratio.

### Patients

To be eligible for COMET [10], patients with severe eosinophilic asthma were required to have received ≥3 years continuous mepolizumab treatment (no break >12 weeks between any two mepolizumab doses) and must have completed either the COLUMBA (MEA115666; NCT01691859) [23] or COSMEX (201312; NCT02135692) [24] studies.

### Endpoints and assessments

The primary endpoint (time to first clinically significant exacerbation) and secondary efficacy endpoints have been reported [10]. Additional health outcome endpoints evaluated during COMET include time to worsening of asthma, global rating of asthma severity, overall perception of response to therapy and unscheduled HCRU.

Time to worsening of asthma was defined as patients meeting at least two of four criteria for at least two consecutive days as captured by electronic diary (eDiary): an increase of ≥50% in rescue medication usage compared with average use during the previous week; an asthma symptom score of 5 (symptoms so severe the patient cannot perform normal daily activities); awakening due to asthma symptoms during the night requiring rescue medication; a decrease in morning peak expiratory flow (PEF) ≥30% compared with COMET baseline.

The global rating of asthma severity and overall perception of response to therapy were performed by clinicians and patients prior to any other procedures during each study visit. The global rating of asthma severity was a single question asking patients and clinicians to rate the patient's asthma severity as mild, moderate, severe or very severe (1–4-point rating scale, respectively) measured at baseline and Weeks 12, 24, 36 and 52. Overall perception of response to therapy evaluated the patient's response to treatment at Weeks 12, 24, 36 and 52 compared with the patient's asthma prior to initiating double-blind treatment at the first visit of Part C using a 7-point rating scale (1: significantly improved to 7: significantly worse, detailed in supplementary materials) and was completed separately by the clinician and patient.

Unscheduled asthma-related HCRU included all unscheduled healthcare contacts, unscheduled visits and time off work/school due to asthma (detailed in supplementary materials).

### Sample size and statistical analysis

Sample size calculations were based on the primary efficacy endpoint and an assumed withdrawal rate [10]. The intent-to-treat population comprised all randomised patients who received one or more doses of double-blind study medication within Part C and was used for all health outcomes endpoint analyses. For all change from baseline endpoints, baseline was defined as the start of double-blind treatment (randomisation) in COMET.

Times to event endpoints were analysed using Kaplan–Meier estimates and Cox proportional hazards models, with adjustment for covariates. Changes from baseline in eDiary endpoints were analysed using mixed model repeated measures. The global rating of asthma severity and overall perception of response to therapy were analysed using a proportional odds model (multinomial (ordered) logistic generalised linear model) with adjustment for covariates. Patients were categorised according to their response.

Further details of the statistical methods used for all endpoints are provided in the supplementary materials.

## Results

### Patient population

Details of the patient populations, including demographics, have been previously reported [10] and were similar between the two treatment groups; further baseline data related to health outcomes endpoints are detailed in table 1. While baseline global rating of asthma severity was similar between treatment arms, there was a difference between how patients and clinicians rated asthma severity, with clinicians more often rating asthma as “severe” or “very severe”. A total of 295 patients progressed into Part C and were randomised 1:1 to receive double-blind treatment of either placebo (n=151) (stopped mepolizumab) or mepolizumab 100 mg SC (n=144) (continued mepolizumab) (supplementary figure 1). In total, 129 patients (n=84 stopped mepolizumab arm; n=45 continued mepolizumab arm) discontinued double-blind treatment and moved into optional Part D following an asthma exacerbation, as permitted by the study design (supplementary figure 1).

**TABLE 1:** Health outcomes and baseline demographics of patients following long-term mepolizumab treatment for ≥3 years at first clinic visit during Part C

	**Stopped mepolizumab (N=151)**	**Continued mepolizumab (N=144)**	**Total (N=295)**
**Females, n (%)**	86 (57)	87 (60)	173 (59)
**Age, years, mean (sd)**	55.7 (11.42)	56.6 (11.53)	56.1 (11.46)
**White, n (%)**	125 (83)	115 (80)	240 (81)
**Duration of asthma, years, mean (sd)**	22.8 (13.82)	25.1 (14.54)	23.9 (14.20)
**Blood eosinophil count, cells·µL^−1^, geometric mean (sd of log)**	40 (0.87)	50 (0.88)	50 (0.88)
**Mepolizumab continuous exposure prior to randomisation at Visit C1**			
Time on mepolizumab, months, median (range)	44.1 (36–59)	43.6 (32–58)	44.1 (32–59)
Total exposure, patient-years	588.0	557.2	1145.2
**Rescue use^#^, occasions·day^−1^ BD used, mean (sd)**	0.39 (0.43)	0.50 (0.45)	0.44 (0.44)
**Daily asthma symptom score^#^, mean (sd)^¶^**	1.03 (0.99)	1.11 (1.04)	1.07 (1.02)
**Awakenings at night due to asthma symptoms requiring rescue medication use^#^, mean (sd)**	0.29 (0.55)	0.40 (0.73)	0.34 (0.64)
**Morning PEF^#^, L·min^−1^, mean (sd)**	307 (124)	296 (117)	301 (121)
**Global rating of asthma severity, n (%)**			
Clinician			
n	148	144	292
Mild	25 (17)	18 (13)	43 (15)
Moderate	60 (41)	56 (39)	116 (40)
Severe	52 (35)	61 (42)	113 (39)
Very severe	11 (7)	9 (6)	20 (7)
Patient			
n	151	144	295
Mild	53 (35)	38 (26)	91 (31)
Moderate	76 (50)	72 (50)	148 (50)
Severe	18 (12)	28 (19)	46 (16)
Very severe	4 (3)	6 (4)	10 (3)

BD: bronchodilator; PEF: peak expiratory flow. ^#^Information collected on a daily basis using an eDiary and averaged over the 4-week period prior to the first dose of double-blind treatment. ^¶^Asthma symptom score evaluated using a 6-point scale of 0 (no symptoms) to 5 (symptoms so severe unable to perform normal daily activities).

### Asthma worsening

Analysis of time to first asthma worsening during Part C demonstrated a 71% increased risk for patients who stopped mepolizumab relative to those who continued mepolizumab, over the 52-week treatment period (hazard ratio (HR) 1.71; 95% CI 1.17–2.52; p=0.006) (figure 1). Separation of asthma worsening was evident from Week 12; fewer patients continuing mepolizumab (16.2%) experienced a worsening of asthma compared with those who stopped (26.1%), which was maintained until Week 52.

**FIGURE 2:**
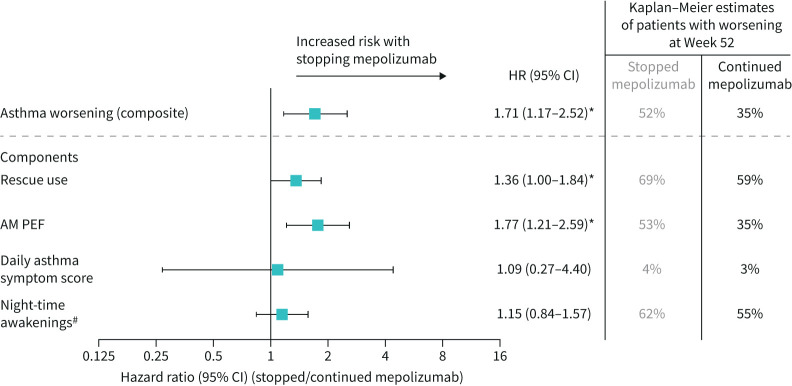
Risk of asthma worsening (composite and individual components) over 52 weeks for patients who stopped mepolizumab *versus* those who continued. *p<0.05. ^#^Due to asthma symptoms requiring rescue medication use. Asthma worsening defined as patients meeting at least two of the possible four eDiary criteria for at least two consecutive days. For individual components: patients meeting the eDiary criterion for at least two consecutive days were considered as experiencing a worsening in asthma. Hazard ratio estimated using a Cox proportional hazards model with terms for treatment group and adjustment for baseline covariates. AM: morning; HR: hazard ratio; PEF: peak expiratory flow.

The two parameters that contributed to asthma worsening in patients who stopped mepolizumab were increased rescue use and worsening of morning PEF over time (supplementary figure 2, panels A, B). This culminated in higher rescue medication use and decreased morning PEF at Week 52 for patients who stopped *versus* continued mepolizumab, and an estimated increased risk of first worsening in rescue use and morning PEF (HR 1.36; 95% CI 1.00–1.84; p=0.047 and HR 1.77; 95% CI 1.21–2.59; p=0.003, respectively) (figure 2). Of the remaining parameters, there was minimal impact in the time to first worsening in daily asthma symptom score and awakening at night in patients who stopped *versus* continued mepolizumab (figure 2 and supplementary figure 2, panels C, D).

When measuring change from COMET baseline in daily asthma symptom scores and morning PEF, the mean change worsened at all time points in patients who stopped mepolizumab. Patients who continued mepolizumab experienced sustained benefit in these outcomes (mean change of approximately zero at all time points) (supplementary figure 3, panels A, B) with a significant difference between treatment arms at Week 52 (daily asthma symptom score p=0.013; morning PEF p≤0.001). Although improvements were seen in change from COMET baseline rescue use and awakening at night for both groups, there was no significant difference between treatment arms (supplementary figure 3, panels C, D).

**FIGURE 3:**
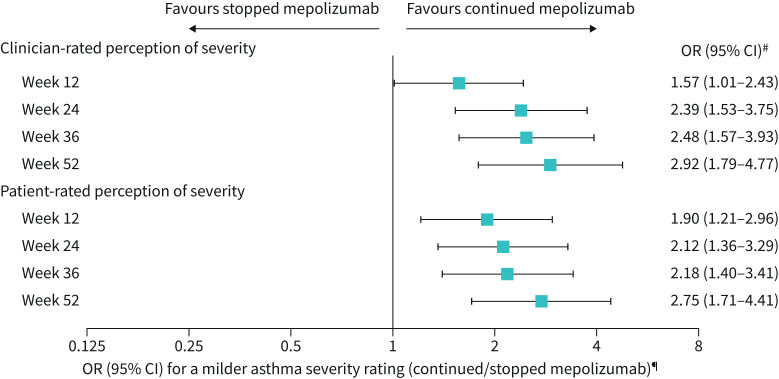
Global rating of asthma severity. Perceived asthma severity was rated on a 4-point scale: “mild”, “moderate”, “severe”, “very severe”. Three patients with missing clinician-rated global rating of asthma severity at baseline were not included in the analysis of clinician-rated perceptions of severity. Data were classified as missing when a rating was not given/obtained or when patients were switched back to open-label mepolizumab treatment (Part D). ^#^p<0.001 for all time points except Week 12 (p=0.045 for clinician; p=0.005 for patient). ^¶^A milder asthma severity rating indicates a less severe response on this rating scale at the given time point. Analysed using a proportional odds model (multinomial (ordered) logistic generalised linear model), with terms for treatment group and adjustment for baseline covariates. For analysis purposes, very severe and missing were combined into one category.

### Global rating of asthma severity

At COMET baseline, the proportion of patients evaluated as “severe” or “very severe” by either clinician or patient was marginally higher in patients who continued mepolizumab (clinician: stopped mepolizumab 63 (42%) *versus* continued mepolizumab 70 (49%), patient: 22 (15%) *versus* 34 (24%), table 1).

Patients were significantly more likely to have milder asthma severity ratings at all time points (p<0.001 for all time points except Week 12 (p=0.045 for clinician; p=0.005 for patient)) when continuing mepolizumab compared with patients who stopped mepolizumab as rated by both clinicians and patients (figure 3). The proportion of patients who experienced a worsening of asthma severity or missing evaluation during Part C was higher at all time points for patients who stopped *versus* continued mepolizumab as assessed by both clinicians and patients. The proportion of patients who experienced no change or an improvement from baseline was generally lower for patients who stopped *versus* continued mepolizumab (supplementary figure 4).

**FIGURE 4:**
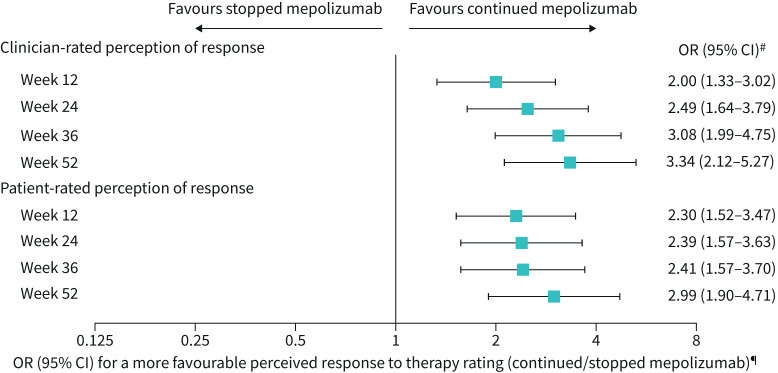
Overall perception of response to therapy. The overall evaluation of perceived response to therapy was rated on a 7-point scale and compared with baseline. Responses included: 1=significantly improved; 2=moderately improved; 3=mildly improved; 4=no change; 5=mildly worse; 6=moderately worse; 7=significantly worse. Data were classified as missing when a rating was not given/obtained or when patients were switched back to open-label mepolizumab treatment (Part D). ^#^p<0.001 for all time points. ^¶^A more favourable response to therapy rating indicates a response in the direction of improvement on this rating scale at the given time point. Analysed using a proportional odds model (multinomial (ordered) logistic generalised linear model), with terms for treatment group and adjustment for baseline covariates. For analysis purposes, significantly worse and missing were combined into one category.

### Overall perception of response to therapy

Patients who continued mepolizumab were significantly more likely to perceive a more positive/favourable response to therapy compared with patients who stopped mepolizumab (p<0.001 for all time points) (figure 4), as assessed by both clinicians and patients. The proportion of patients that experienced a worsening perception of response to therapy (mildly, moderately or significantly worse) or a missing evaluation was higher for patients who stopped mepolizumab at all time points than for patients who continued mepolizumab, whether patient- or clinician-rated (supplementary figure 5). The proportion of patients with improved (mildly, moderately or significantly improved) or no change in evaluation score were both generally lower for patients who stopped mepolizumab *versus* patients who continued mepolizumab (supplementary figure 5).

**FIGURE 5:**
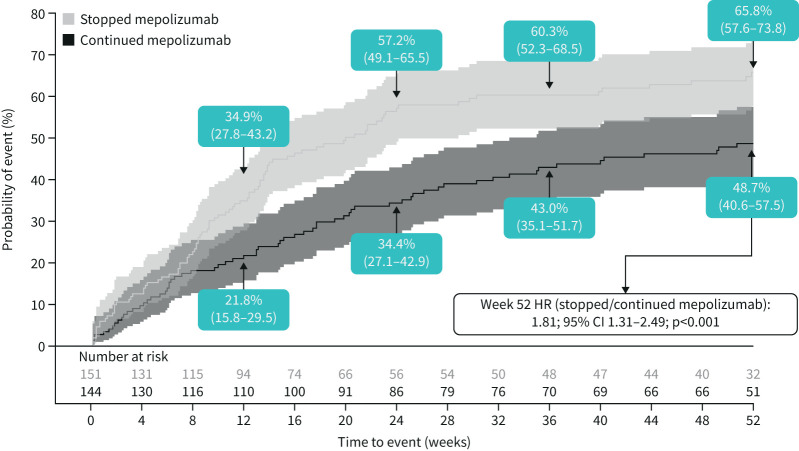
Time to first unscheduled healthcare resource use. Kaplan–Meier cumulative incidence curves for time to first unscheduled healthcare resource use due to asthma (including all unscheduled healthcare resource use of any type (as detailed in supplementary materials) and any time off work/school). Values in boxes indicate the Kaplan–Meier estimates of the probability of healthcare resource use within the 95% CI (also reflected by shaded intervals). HR estimated using a Cox proportional hazards model with terms for treatment group and adjustment for baseline covariates. HR: hazard ratio.

### Unscheduled HCRU

Overall, stopping mepolizumab led to a higher probability of any unscheduled HCRU (including all unscheduled HCRU of any type (detailed in supplementary materials) and any time off work/school due to asthma) compared with continuing mepolizumab, for all time points measured (figure 5) (HR 1.81; 95% CI 1.31–2.49; p<0.001). By Week 52, 65.8% *versus* 48.7% of patients who stopped *versus* continued mepolizumab had experienced unscheduled HCRU. When the component parts of unscheduled HCRU (unscheduled visits (including urgent care/outpatient visits or hospitalisation/emergency room visits), having time off work or school) were analysed separately, patients in both groups had a similar risk (Week 52: HR 1.09; 95% CI 0.57–2.08; p=0.788 and HR 1.22; 95% CI 0.75–2.00; p=0.427, respectively), although the number of unscheduled visits was low.

## Discussion

These analyses support and extend findings from the primary manuscript [10], demonstrating that benefits gained from long-term mepolizumab therapy in patients with severe eosinophilic asthma are not maintained after stopping mepolizumab treatment. Discontinuing mepolizumab in patients who have responded to mepolizumab for at least 3 years led to day-to-day patient-reported asthma worsening, an increase in patient and clinician perceived disease severity, as well as reduced perceived response to therapy and increased unscheduled HCRU. Of note, this worsening was observed by Week 12 (16 weeks after each patient's last dose of open-label mepolizumab), which follows the previously reported increase in blood eosinophil counts that occurred 8 weeks after the last dose of mepolizumab, reflecting the mechanism of action of mepolizumab [10].

There was an increased risk and shorter time to first asthma worsening for patients stopping mepolizumab compared with those continuing, largely driven by worsening in rescue medication use and morning PEF. Indeed, morning PEF was significantly worse in patients who stopped *versus* continued mepolizumab from Week 4, which may be an early indication of declining lung function. These data suggest that it may be beneficial to monitor daily morning PEF if a patient stops mepolizumab therapy, to provide an early indication of asthma worsening, although this requires additional analysis to confirm. When assessed in relation to time to first asthma worsening, asthma symptom score was not sensitive due to the low number of patients who reported the worst possible asthma symptom score in either treatment group; however, there was a clear treatment difference between groups in terms of mean change. The worsening observed in mean change of daily asthma symptom score and morning PEF for patients stopping mepolizumab are consistent with other outcomes reported previously [10], including asthma control (measured by Asthma Control Questionnaire-5 score), HRQoL (measured by St George's Respiratory Questionnaire score) and lung function (pre-bronchodilator forced expiratory volume in 1 s) results. The outcomes described here hold clinical significance and reflect the daily burden of patients with severe eosinophilic asthma; these are important measures that reflect how it feels as a patient to have severe asthma day to day, not just at the threshold of an exacerbation. These data contrast with patients continuing mepolizumab, where these outcomes remained unchanged, with mean change from baseline of approximately zero at all time points. Generally, these results parallel changes previously reported in blood eosinophil count [10], with those patients stopping mepolizumab experiencing significant increases by Week 12 compared with patients continuing mepolizumab. These analyses further confirm that stopping mepolizumab leads to loss of asthma control, and the benefits of mepolizumab therapy are maintained when patients continue treatment.

Patients who continued mepolizumab reported milder asthma severity and a more favourable perceived response to therapy compared with patients who stopped mepolizumab, a finding mirrored by clinician responses. This indicates a longevity in the improvement of HRQoL, which has previously been reported in the MUSCA and COMET studies [10, 21].

Patients who stopped mepolizumab had an increased risk of unscheduled HCRU, with the incidence of events largely aligning to the previously reported incidence of time to first exacerbation (stopping/continuing mepolizumab HR 1.61; 95% CI 1.17–2.22; p=0.004) [10]. Interestingly, the proportion of patients at risk of an unscheduled HCRU was higher than those experiencing an exacerbation, suggesting that healthcare usage is not captured by exacerbation analysis alone. There was no difference in either time to first unscheduled visit or time to first time off work/school when analysed independently for patients who stopped *versus* continued mepolizumab, which may in part be due to low numbers of events. Time to first unscheduled visit mirrors similarly low numbers of patients experiencing exacerbations requiring hospitalisation/emergency room visits detailed in the primary manuscript [10]. The low number of these visits may reflect that patients were switched to open-label mepolizumab prior to a more severe exacerbation.

Overall, the results from COMET support previous studies that demonstrated the clinical benefit and durability of effect of long-term mepolizumab treatment in severe eosinophilic asthma [23, 24]. While COMET is the first study to examine stopping mepolizumab after previous long-term treatment, earlier studies have shown that long-term treatment with mepolizumab induces and maintains significant reductions in exacerbation rates and OCS use compared with placebo [19, 20, 22, 24], and these beneficial effects can be recovered after a break in treatment [23]. Interestingly, the rapid worsening of morning PEF seen here in patients stopping mepolizumab aligns with post hoc analysis performed on the MENSA (NCT01691521) and MUSCA (NCT02281318) studies [26]; rapid improvement in morning PEF (from Weeks 1–4) was observed in these studies following mepolizumab therapy initiation, supporting the concept that changes in morning PEF may be a useful early predictive marker for therapeutic response with mepolizumab treatment, although additional studies are needed.

In combination with the data here, these studies suggest that even after long-term treatment with mepolizumab, maintaining therapy is imperative to sustain clinical benefits perceived by both patients and clinicians. Similar to other asthma treatments such as omalizumab, where withdrawal of treatment leads to loss of clinical benefits [27], long-term mepolizumab does not initiate disease remission in this patient population. Of note, some patients in the current study who stopped mepolizumab maintained asthma control, a finding that has been reported with previous mepolizumab studies [18, 19], and other asthma treatments where a proportion of the placebo group maintained asthma control [27]. This observation likely reflects the reported variability of the disease phenotype [1, 3–5] and/or may reflect that patients used their standard medications more consistently in a trial setting, or avoided other factors that contribute to asthma worsening. Determining indicators of whether a patient can stop treatment, temporarily or permanently, and maintain asthma control, is of paramount importance for the management of patient care. In contrast, some patients who remained on long-term mepolizumab did not maintain asthma control, with a subset showing asthma worsening. For patients with severe eosinophilic asthma, variability in response to mepolizumab treatment, and in which aspects of disease indicate treatment benefit (*e.g.* PEF, night-time awakenings or rescue medication use), has been previously reported [18, 19, 21, 22]. Therefore, analysis of patient subgroups based on response to therapy [28] or rate of asthma worsening [29] may allow insights into potential disease course and optimal treatment for patients. Further investigation and stratified analysis of different subsets of patients within this study are an ongoing focus [10].

One strength of the current study is the monitoring and maintenance of background therapy throughout Part C, with no changes recommended within the protocol. There were also several potential limitations of the study. Patients were selected from a population who had completed previous mepolizumab clinical trials; therefore, patient recruitment may have been biased toward those who may have responded to mepolizumab, and those without adverse events during previous studies. As such, these results would need confirmation in a real-world clinical setting. More patients who stopped mepolizumab treatment switched to open-label mepolizumab (Part D) following an asthma exacerbation in Part C, compared with patients who continued mepolizumab. Due to the application of a composite estimand strategy during analysis of the global rating of asthma severity and overall perception of response to therapy, all patients who switched to Part D were classed as treatment failures for all later time points, meaning that more patients in the stopped mepolizumab arm were assigned the least favourable response. Furthermore, due to large numbers of patients discontinuing Part C and switching to Part D, some results at later time points are based on lower numbers of patients. However, an increased prevalence for switching to open-label treatment in the placebo group supports the benefits of continued mepolizumab treatment. Finally, while the patient and clinician assessed global rating of asthma severity and overall perception of response to therapy endpoints provide valuable patient and clinician reported information on the effects of stopping *versus* continuing mepolizumab treatment, it should be noted that the tools used have not been formally validated in a patient population with severe asthma.

Overall, following long-term treatment with mepolizumab, patients who stopped mepolizumab experienced a higher risk of asthma worsening and reduced disease control *versus* those who continued therapy. Additionally, both clinicians and patients perceived less clinical benefit when stopping compared with continuing mepolizumab and patients required higher levels of unscheduled healthcare resources related to asthma. The data presented here, in conjunction with the primary COMET results, suggest that for patients with severe eosinophilic asthma, continued mepolizumab treatment sustains clinically important improvements in health outcomes perceived by both patients and clinicians, beyond 3 years, and that morning PEF could be a potential early predictive marker of loss of therapeutic response.
